# Distribution and drug sensitivity of pathogenic bacteria in diabetic foot ulcer patients with necrotizing fasciitis at a diabetic foot center in China

**DOI:** 10.1186/s12879-022-07382-7

**Published:** 2022-04-22

**Authors:** Xuemei Li, Zhipeng Du, Ziwei Tang, Qin Wen, Qingfeng Cheng, Yunhua Cui

**Affiliations:** 1 Department of Pharmacy, Chongqing Health Center for Women and Children, Chongqing, China; 2 Department of Pharmacy, The First Affiliated Hospital of Chongqing Medical University, Chongqing, China; 3 Department of Endocrinology, The First Affiliated Hospital of Chongqing Medical University, No. 1, Yixueyuan Road, Yuzhong District, Chongqing, 400016 China; 4 Department of Pharmacy, Chongqing Yunyang County Traditional Chinese Medicine Hospital, Chongqing, China; 5 Department of Respiratory and Critical Care Medicine, Xiangyang Central Hospital, Affiliated Hospital of Hubei University of Arts and Science, No. 136, Jingzhou Street, Xiangcheng District, Hubei, 441021 China

**Keywords:** Necrotizing fasciitis, Diabetic foot ulcer, Pathogenic bacteria, Antimicrobial susceptibility test, Initial empirical antimicrobials

## Abstract

**Background:**

Diabetic foot ulcer is one of the major complications for patients with diabetes, and has become an important cause of non-traumatic amputation. Necrotizing fasciitis is a life-threatening soft-tissue infection involving the fascia and subcutaneous tissue. When diabetic foot ulcers are complicated by necrotizing fasciitis (DNF), this increases the risk for amputation and mortality, making DNF treatment more complicated, and eventually leading to amputation and mortality. However, studies on pathogenic bacteria’s distribution and drug sensitivity in DNF patients remain lacking. This study investigated the distribution and susceptibility of pathogenic bacteria in DNF patients, and provided empirical antibacterial guidance for the clinic.

**Methods:**

In a single diabetic foot center, the results from microbial cultures and drug susceptibility tests of patients with DNF from October 2013 to December 2020 were collected and analyzed.

**Results:**

A total of 101 DNF patients were included in this study, of whom 94 had positive culture test results. A total of 124 pathogens were cultured, including 76 Gram-positive bacterial strains, 42 Gram-negative bacterial strains, and six fungal strains. Polymicrobial infections accounted for 26.7% and monomicrobial infections accounted for 66.3%. *Staphylococcus aureus* was the most common bacterium isolated, followed by *Enterococcus faecalis* and *Streptococcus agalactiae*. *Pseudomonas aeruginosa*, *Klebsiella pneumoniae*, and *Proteus mirabilis* were the most common Gram-negative bacteria. Thirty-five strains of multi-drug resistant bacteria were isolated, representing 28.2% of the total isolates. Gram-positive bacteria were more sensitive to levofloxacin, moxifloxacin, vancomycin, teicoplanin, tigecycline, and linezolid, while Gram-negative bacteria were more sensitive to amikacin, piperacillin/tazobactam, cefoperazone/sulbactam, ceftazidime, cefepime, imipenem, and meropenem.

**Conclusions:**

Gram-positive bacteria were the main bacteria isolated from DNF patients. The bacterial composition, the proportion of multi-drug resistant bacteria among the pathogens, and the high risk for amputation should be fully considered in the initial empirical medication, and broad-spectrum antibacterials are recommended.

## Background

Diabetic foot ulcer (DFU) is one of the major complications for patients with diabetes, and has become an important cause of non-traumatic amputation [1–4]. Most patients with DFUs display peripheral vascular disease or diabetic neuropathy, and have impaired immunity [5, 6]. These conditions reduce the penetration of antimicrobial drugs in DFUs, making it difficult to reach the target site and achieve effective antimicrobial action, and would be able to rapidly induce bacterial resistance. Necrotizing fasciitis (NF) is a relatively rare soft-tissue infection involving the fascia and subcutaneous tissue [7–11]. This disease develops rapidly, such that clinically, early recognition and timely surgical incision and drainage decompression are needed, otherwise it can readily lead to multiple organ failure and septic shock, even becoming life-threatening [11, 12], with a mortality rate as high as 30–35% [13]. NF occurs more frequently in diabetes and the consequences are more serious [7, 13–18]. Therefore, when DFU is complicated by NF (DNF), this makes treatment more complicated with a poor prognosis, thus, increasing the average hospital stay and causing a major economic burden through an increased risk for amputation and mortality.

Consequently, DNF requires prompt and aggressive surgical treatment, as well as appropriate antibiotic therapy [19, 20]. However, studies on the distribution and drug sensitivity of pathogenic bacteria in DNF patients remain lacking. In addition, the NF infection in the foot differs from other parts of the body because of physiological changes (neuropathy and peripheral arterial disease), such that the treatment in the foot is more complicated than in other bodily regions. We found only one study that reported the microbial distribution in DNF patients, but without drug susceptibility results [17]. In other studies, the location of diabetes with NF was not limited to the feet, including limbs [19] or all bodily regions [21, 22].

In this study, we retrospectively investigated the distribution and drug susceptibility of pathogenic bacteria in DNF patients, to help clinicians choose a more appropriate empiric antimicrobial regimen for DNF. To our knowledge, this is the first report on the sensitivity of pathogenic bacteria in DNF patients.

## Methods

### Patients

**Fig. 1:**
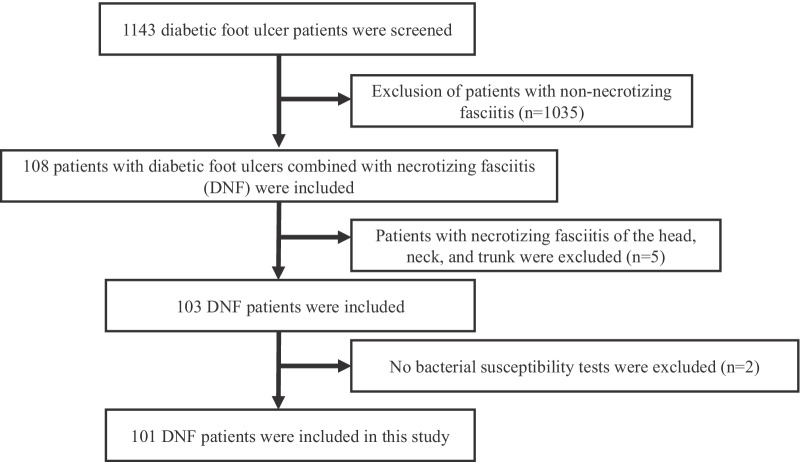
The flow chart of patients who met inclusion and exclusion criteria for the study population

A total of 101 patients with DNF admitted to the Department of Endocrinology of the First Affiliated Hospital of Chongqing Medical University from October 2013 to December 2020, were retrospectively evaluated in this study (Fig. 1). All patients diagnosed with DFUs met the diagnostic criteria of the 2020 International Working Group on the Diabetic Foot (IWGDF) guidelines [23]. All patients with NF met the following diagnostic criteria for NF proposed by Fisher et al. and were confirmed as NF by surgery [10]: (1) extensive necrosis of the superficial fascia with widespread destruction of surrounding tissue; (2) absence of muscle involvement; (3) no clostridium was present in wound and blood cultures; (4) absence of major vascular occlusion; and (5) extensive leukocyte infiltration, focal necrosis of fascia and adjacent tissues, and microvascular thrombosis were found by the pathological examination of debridement tissue. All patients underwent surgery to clear necrotic tissue (Table 1). Indications for surgery were: patients with skin and soft tissue infections that had an acute onset and short course, local tissues and skin had redness, swelling, abnormal pain, numbness, and blisters or bloody blisters, and local physical examination indicated a possible sense of fluctuation or subcutaneous crepitus, and the finger test was positive (there was a loss of feeling between the skin and the fascia, suggesting possible necrosis of the fascia) [24, 25]. Patients with other infectious diseases (pneumonia and urinary tract infection) or no bacterial susceptibility tests were excluded from the study. Patients with necrotizing fasciitis of the head, neck, and trunk were also excluded.

**Table 1:** Inclusion and Exclusion criteria for selecting DNF patients

Inclusion criteria	Exclusion criteria
1. All patients diagnosed with DFUs met the diagnostic criteria of the 2020 International Working Group on the Diabetic Foot (IWGDF) guidelines 2. All patients with NF met the following diagnostic criteria: 1) extensive necrosis of the superficial fascia with widespread destruction of surrounding tissue; 2) absence of muscle involvement; 3) no clostridium was present in wound and blood cultures; 4) absence of major vascular occlusion; 5) pathological examination of debridement tissue: extensive leukocyte infiltration, focal necrosis of fascia and adjacent tissues, and microvascular thrombosis 3. All patients underwent surgery to clear necrotic tissue	1. Patients with other infectious diseases; 2. No bacterial susceptibility tests were excluded; 3. Patients with necrotizing fasciitis of the head, neck, trunk, or other sites were excluded

### Specimen collection

Swab specimens were collected from the ulcer base of the DNF patients free from antibiotics. To avoid isolation of colonizing bacteria, the ulcer wound was flushed with saline solution and the necrotic tissue and exudates on the surface were removed. Swabs were immediately scrubbed and rolled in a “Z” pattern onto the ulcer base by nurses and then were put into sterile test tubes [26]. Soft tissue specimens were obtained when the plastic surgeon cleaned up the necrotic tissue during operation and debridement. The number of swab samples collected per patient on admission was 3 swab specimens, and the number of samples sent for surgical debridement was one piece of tissue. When soft tissue susceptibility results were available for DNF patients, these results were prioritized as the susceptibility results of the DNF patients in this study. If unavailable, the swab susceptibility results were regarded as the susceptibility results. If soft tissue and swab susceptibility results were not available, the blood culture results were used as the susceptibility results.

### Microbiological assessment

Bacterial isolates were identified by VITEK-MS mass spectrometer and VITEK2-Compact instrument produced by Bio Mérieux, France. The drug susceptibility testing was detected by Kirby- Bauer method, automatic microbiological analyzer (VITEK2-Compact) and E test method. The bacterial identification card and drug susceptibility card used were produced by Bio-Merieux Company, Mueller–Hinton agar for drug sensitivity testing by Kirby-Bauer method was produced by Thermo Fisher Company, and the drug susceptibility disk was produced by Oxoid Company. All drug susceptibility results were interpreted in accordance with the performance standards for antimicrobial drug susceptibility testing by the American Clinical and Laboratory Standardization Institute (CLSI) in 2020. Multi-drug resistant (MDR) strains were evaluated according to the standard definitions of multidrug-resistant and extensively drug-resistant bacteria published by Magiorakos [27].

### Statistical analyses

All data were analyzed by SPSS 22.0 software. The continuous variables were expressed as mean ± standard deviation (x ± s). The values and ratios of the qualitative variables were counted.

## Results

### Patient and ulcer characteristics

We included a total of 101 patients with DNF, including 67 (66.3%) men and 34 (33.7%) women. There were 98 patients (97.0%) had type 2 diabetes with a mean age of 60.86 ± 11.08, and 3 patients had type 1 diabetes with a mean age of 42.67 ± 17.50 (Table 2). Among the type 2 diabetic, 49 (50.0%) patients were aged over 60 years. The mean duration of diabetes was 10.27 ± 7.09 years, the mean hospital stay was 30.17 ± 21.93 days, the mean blood glucose at admission was 16.37 ± 6.29 mmol/L, and the mean glycated hemoglobin level was 11.46 ± 2.79%. Six patients had good glycemic control (HbA1c ≤ 7.0%). All patients were of Wagner grade 3. The mean wound duration prior to admission was 44.29 ± 76.96 days. A total of 44 people (43.6%) used insulin before admission, with the most used types of insulin being premixed insulin (21.8%), intermediate-acting insulin (6.9%) and rapidly acting insulin analogs + basal insulin analogs (5.0%). The foot ulcers in all patients were not in the same location, and the most common sites were the planta, foot dorsum, hallux, and the fifth phalanx. There were 45 concomitant diseases (the concomitant diseases were not the same in different patients), the most common concomitant diseases being hypertension (45.5%), anemia (25.7%), hypoproteinemia (24.8%), osteoporosis (19.8%) and lower extremity atherosclerotic occlusive disease (17.8%). A total of 49 (48.5%) patients with DNF displayed vascular disease, 95 (94.1%) had peripheral neuropathy, 43 (42.6%) had renal dysfunction, and 54 (53.5%) had retinopathy. Amputation was performed on 32 patients (20%). A total of 73 (72.3%) patients received antibiotics one month before admission.

**Table 2:** Demographical and clinical characteristics of 101 diabetic foot patients with necrotizing fasciitis

Variables	Number (%)
Male	67 (66.3%)
Female	34 (33.7%)
Age	60.32 ± 11.62
Type 1 Diabetes	3 (3.0%)
Type 2 Diabetes	98 (97.0%)
The duration of diabetes, years	10.27 ± 7.09
Blood Glucose (mmol/l)	16.37 ± 6.29
HbA1c (%) ≤ 7% (good control)	11.46 ± 2.79 6 (5.8%)
Hospital stays, days	30.17 ± 21.93
Duration of the ulcer, days	
30 days or less	76 (75.2%)
Over 30 days	25 (24.8%)
The type of insulin	
Pre-mixed insulins	22 (21.8%)
Intermediate-acting insulin (NPH)	7 (6.9%)
Rapidly acting insulin analogs + Basal insulin analogs	5 (5.0%)
Rapidly acting insulin analogs	3 (3.0%)
Basal insulin analogs	2 (2.0%)
Rapidly acting insulin analogs + NPH	1 (1.0%)
Type of insulin was not known	4 (4.0%)
Location of foot ulcers	
Planta	31
Foot dorsum	20
Hallux	17
The second phalanx	14
The third phalanx	10
The fourth phalanx	9
The fifth phalanx	26
Others	19
Concomitant disease	
Hypertension	46 (45.5%)
Anemia	26 (25.7%)
Hypoproteinemia	25 (24.8%)
Osteoporosis	20 (19.8%)
Lower extremity atherosclerotic occlusive disease	18 (17.8%)
Complications	
Neuropathy	95 (94.1%)
Lower extremity vascular disease	49 (48.5%)
Nephropathy	43 (42.6%)
Retinopathy	54 (53.5%)
The amputation rate	32 (31.7%)
Antibiotics use before admission	73 (72.3%)

### Distribution of pathogens according to the culture technique

Of the 101 specimens from the DNF patients, 46 were swabs, 53 were tissue specimens, and 2 were blood specimens. As shown in Table 3, a total of 94 (93.1%) patients had positive culture test results. Monomicrobial (type II) necrotizing infection, a single pathogen species was isolated from 67 specimens (66.3%). A total of 27 cases (26.7%) were polymicrobial (type I) necrotizing infections, including two pathogen species isolated from 24 specimens (23.8%), and three pathogen species isolated from three specimens (3.0%). A total of 124 strains of pathogens were isolated, including 76 (61.3%) Gram-positive bacteria and 42 (33.9%) Gram-negative bacteria, while six strains (4.8%) were fungi. The mean number of isolates per specimen was 1.23 (range, 1–3).

**Table 3:** Distribution of pathogenic bacteria in diabetic foot ulcer patients with necrotizing fasciitis

Pathogens	Number (%)
Positive specimens	94 (93.1%)
No. of isolates	124
Mean no. of isolates per specimen	1.23
MDR	35 (28.2%)
Monomicrobial infection	67 (66.3%)
Polymicrobial infection	27 (26.7%)
Gram-positive bacteria	76 (61.3%)
*Staphylococcus aureus*	20 (16.1%)
MRSA	5 (4.0%)
Coagulase-negative staphylococci	7 (5.6%)
*Staphylococcus haemolyticus*	1 (0.8%)
Streptococcus	22 (17.7%)
*Enterococcus*	23 (18.5%)
*Enterococcus faecalis*	18 (14.5%)
Other Gram-positive bacteria	3 (2.4%)
Gram-negative bacteria^a^	42 (33.9%)
*Pseudomonas aeruginosa*	5 (4.0%)
*Klebsiella pneumoniae*	5 (4.0%)
*Proteus mirabilis*	5 (4.0%)
*Morganella morganii*	4 (3.2%)
*Escherichia coli*	3 (2.4%)
*Proteus penneri*	3 (2.4%)
*Enterobacter cloacae*	3 (2.4%)
*Acinetobacter baumannii*	2 (1.6%)
*Raoultella ornithinolytica*	2 (1.6%)
*klebsiella oxytoca*	2 (1.6%)
*Other Gram-negative bacteria*^*b*^	8 (6.5%)
Fungus	6 (4.8%)
*Candida glabrata*	3 (2.4%)
*Candida albicans*	1 (0.8%)
*Candida parapsilosis*	1 (0.8%)
C*andida tropicalis*	1 (0.8%)

^a^Other Gram-positive bacteria refers to 2 strains of *Arcanobacterium haemolyticum* and 1 strain of *Corynebacterium striatum*

^b^Other Gram-negative bacteria refers to *Citrobacter braakii*, *Proteus hauseri, Alcaligenes faecalis*, *Citrobacter freundii*, *Citrobacter koseri*, *Proteus vulgaris*, *Stenotrophomonas maltophilia*, *Serratia marcescens*, each of which had one strain

*Staphylococcus aureus* was the most common pathogen of the total isolated bacteria (16.1%), and the most common Gram-positive bacteria, accounting for 26.3% of such bacteria. *Enterococcus faecalis* and *Streptococcus agalactiae* were the second and third most common Gram-positive bacteria, accounting for 23.7% and 14.5%, respectively. Staphylococci accounted for 22.6% of the total isolated bacteria and 36.8% of the Gram-positive bacteria. Enterococci accounted for 18.5% of the total isolates and 30.3% of the Gram-positive bacteria. Streptococci accounted for 17.7% of the total isolated bacteria and 28.9% of the Gram-positive bacteria. Enterobacteriaceae was the most dominant bacterial group of the Gram-negative bacteria, accounting for 78.6% of such bacteria and 26.6% of the total bacteria isolated. *Klebsiella pneumoniae*, *Proteus mirabilis*, and *Pseudomonas aeruginosa* were the most common Gram-negative bacteria, each accounting for 11.9% of all such bacteria, followed by *Morganella morganii* (9.5%), *Escherichia coli* (7.1%), *Proteus penneri* (7.1%), and *Enterobacter cloacae complex* (7.1%). A total of six fungal strains (4.8%) were isolated, including three strains of *Candida glabrata* (2.4%), one strain of *Candida albicans* (0.8%), one strain of *Candida parapsilosis* (0.8%), and one strain of *Candida tropicalis* (0.8%). One strain of *Candida glabrata* was cultured together with *Klebsiella pneumoniae*, while the other fungi were cultured separately.

### Distribution of MDR bacteria

As shown in Table 3, thirty-five MDR bacterial strains were isolated, and the proportion of MDR bacteria to total pathogens was 28.2%. Gram-positive MDR bacteria accounted for 14.5% of the total Gram-positive bacteria while Gram-negative MDR bacteria accounted for 57.1% of the total Gram-negative bacteria. Of the *S. aureus*, 45% were MDR bacteria, with five strains being methicillin-resistant *S. aureus* (MRSA). A total of 60.0% of the *K. pneumoniae* were MDR bacteria. Of the *E. coli*, 66.7% were MDR bacteria. A total of 4 strains of extended-spectrum beta-lactamase (ESBL)-producing bacteria, including 2 strains of *Klebsiella pneumoniae* and 2 strains of *Escherichia coli*. A total of 80.0% of the *P. mirabilis* were MDR bacteria, as were 75.0% of the *M. morganii* and 66.7% of the *P. penneri*. *E. faecalis* had only one MDR strain, while all three *E. cloacae complex* strains were MDR bacteria.

### Antibacterial susceptibility of pathogens

As shown in Table 4, *S. aureus* was most susceptible to linezolid, quinupristin/dalfopristin, sulfamethoxazole, teicoplanin, tigecycline, vancomycin, nitrofurantoin, daptomycin, and ceftaroline, with a sensitivity of 100%, followed by levofloxacin, moxifloxacin, and rifampicin, with a sensitivity of 90%, and relatively sensitive to ciprofloxacin (82.4%). *E. faecalis* was most susceptible to nitrofurantoin, linezolid, teicoplanin, tigecycline, vancomycin, and daptomycin, with a sensitivity of 100%, followed by ampicillin (94.4%), penicillin G (94.4%), levofloxacin (88.9%), and moxifloxacin (88.9%). *Enterococcus avium* was most susceptible to streptomycin, ciprofloxacin, levofloxacin, moxifloxacin, linezolid, teicoplanin, tigecycline, and vancomycin, with a sensitivity of 100%.

**Table 4:** Antimicrobial susceptibility of Gram-positive bacteria from DNF patients (%)

Antibiotics	Staphyloco-ccus aureus (n = 20)	*Enterococcus faecalis* (n = 18)	*Streptococcus agalactiae* (n = 11)	Streptococcus dysgalactiae (n = 6)	Streptococcu-s anginosus (n = 5)	Enterococ-cus avium (n = 3)	Enterococcus gallinarum (n = 1)	Enterococcus raffinosus (n = 1)
Gentamicin	85.0%	55.6%	–	–	–	66.7%	0.0%	100.0%
Streptomycin	–	75.0%	–	–	–	100.0%	0.0%	100.0%
Ampicillin	–	94.4%	100.0%	–	–	33.3%	0.0%	0.0%
Levofloxacin	90.0%	88.9%	63.6%	100.0%	100.0%	100.0%	0.0%	100.0%
Moxifloxacin	90.0%	88.9%	72.7%	–	–	100.0%	0.0%	100.0%
Ciprofloxacin	82.4%	68.7%	66.7%	–	–	100.0%	0.0%	100.0%
Clindamycin	35.0%	0.0%	22.2%	33.3%	25.0%	0.0%	0.0%	0.0%
Erythrocin	35.0%	5.6%	16.7%	33.3%	25.0%	66.7%	0.0%	100.0%
Macrodantin	100.0%	100.0%	100.0%	–	–	–	0.0%	–
Penicillin G	5.0%	94.4%	100.0%	100.0%	100.0%	66.7%	0.0%	0.0%
Oxacillin	75.0%	–	–	–	–	–	–	–
Quinupristin/Dalfopristin	100.0%	0.0%	100.0%	100.0%	100.0%	50.0%	0.0%	0.0%
Rifampicin	90.0%	–	–	–	–	–	–	–
Sulfamethox-azole	100.0%	0.0%	–	–	–	0.00%	–	–
Tetracycline	64.7%	12.5%	22.2%	50.0%	0.0%	0.0%	0.0%	0.0%
Teicoplanin	100.0%	100.0%	–	–	–	100.0%	–	–
Tigecycline	100.0%	100.0%	100.0%	–	–	100.0%	100.0%	100.0%
Vancomycin	100.0%	100.0%	100.0%	100.0%	100.0%	100.0%	100.0%	100.0%
Linezolid	100.0%	100.0%	100.0%	100.0%	100.0%	100.0%	100.0%	100.0%
Chloramphe-nicol	–	–	–	100.0%	100.0%	–	–	–
Cefotaxime	–	–	–	100.0%	100.0%	–	–	–
Daptomycin	100.0%	100.0%	100.0%	–	–	–	–	–
Ceftaroline	100.0%	–	–	–	–	–	–	–

The antimicrobial agent in the antimicrobial susceptibility testing changes from year to year, so the sensitivity rate is calculated based on the number of bacteria that have actually tested the antimicrobial agent

As shown in Table 5, among the Gram-negative bacteria, all Enterobacteriaceae were 100% sensitive to meropenem and imipenem. Ertapenem (96.9%), piperacillin/tazobactam (93.9%), amikacin (93.3%), and cefoperazone/sulbactam (92.9%) also displayed very strong antimicrobial activity, and all Enterobacteriaceae, except for one strain of ESBL-producing *E. coli*, were sensitive to ertapenem. *P. mirabilis* was 100% sensitive to the antibacterial drugs carbapenems, amikacin, aztreonam, ceftazidime, cefoperazone/sulbactam, cefotetan, piperacillin/tazobactam, and piperacillin, followed by levofloxacin (80.0%). *K. pneumoniae* was 100% sensitive to carbapenems, amikacin, cefotetan, cefoxitin, and tigecycline, and relatively sensitive to cefepime (80.0%), levofloxacin (80.0%), piperacillin/tazobactam (80.0%), cefoperazone/sulbactam (75.0%), and gentamicin (75.0%). *M. morganii* was 100% sensitive to aztreonam, ceftazidime, cefoperazone/sulbactam, cefotetan, cefepime, carbapenems, and piperacillin/tazobactam. *P. aeruginosa* was most susceptible to amikacin, ceftazidime, aztreonam, cefoperazone/sulbactam, ciprofloxacin, levofloxacin, cefepime, gentamicin, imipenem, meropenem, tobramycin, piperacillin/tazobactam, and piperacillin, with a sensitivity of 100%.

**Table 5:** Antimicrobial susceptibility of Gram-negative bacteria from DNF patients (%)

Antibiotics	*Pseudomonas aeruginosa* (n = 5)	*Klebsiella pneumoniae* (n = 5)	*Proteus mirabilis* (n = 5)	*Morganella morganii*(n = 4)	*Escherichia coli* (n = 3)	*Proteus penneri* (n = 3)	Enterobacter cloacae (n = 2)	Acinetobacter baumannii (n = 2)	Raoultella ornithinol-ytica (n = 2)	Klebsiella oxytoca (n = 2)
Ampicillin	0.0%	0.0%	40.0%	0.0%	33.3%	–	–	0.0%	0.0%	0.0%
Ampicillin/Sulbactam	0.0%	50.0%	50.0%	0.0%	33.3%	33.3%	0.0%	100.0%	0.0%	100.0%
Piperacillin/Tazobactam	100.0%	80.0%	100.0%	100.0%	100.0%	100.0%	100.0%	100.0%	100.0%	100.0%
Piperacillin	100.0%	–	100.0%	–	–	–	–	50.0%	–	–
Gentamicin	100.0%	75.0%	25.0%	66.7%	66.7%	66.7%	100.0%	100.0%	100.0%	100.0%
Amikacin	100.0%	100.0%	100.0%	75.0%	66.7%	100.0%	100.0%	100.0%	100.0%	100.0%
Tobramycin	100.0%	50.0%	50.0%	66.7%	100.0%	66.7%	0.0%	100.0%	100.0%	100.0%
Ciprofloxacin	100.0%	40.0%	40.0%	50.0%	33.3%	100.0%	100.0%	100.0%	50.0%	100.0%
Levofloxacin	100.0%	80.0%	80.0%	75.0%	33.3%	100.0%	100.0%	100.0%	100.0%	100.0%
Cefazolin	0.0%	20.0%	40.0%	0.0%	33.3%	0.0%	–	–	0.0%	0.0%
Cefuroxime	0.0%	100.0%	66.7%	0.0%	–	0.0%	50.0%	0.0%	100.0%	100.0%
Cefuroxime Axetil	0.0%	100.0%	50.0%	0.0%	–	–	0.0%	0.0%	100.0%	100.0%
Ceftriaxone	0.0%	60.0%	60.0%	75.0%	33.3%	66.7%	100.0%	100.0%	100.0%	100.0%
Ceftazidime	100.0%	60.0%	100.0%	100.0%	66.7%	100.0%	100.0%	100.0%	100.0%	100.0%
Cefepime	100.0%	80.0%	60.0%	100.0%	66.7%	100.0%	100.0%	100.0%	100.0%	100.0%
Cefotetan	0.0%	100.0%	100.0%	100.0%	66.7%	100.0%	0.0%	0.0%	100.0%	100.0%
Cefoxitin	–	100.0%	66.7%	66.7%	66.7%	100.0%	0.0%	–	50.0%	100.0%
Aztreonam	100.0%	60.0%	100.0%	100.0%	66.7%	100.0%	100.0%	0.0%	100.0%	100.0%
Imipenem	100.0%	100.0%	100.0%	100.0%	100.0%	100.0%	100.0%	100.0%	100.0%	100.0%
Ertapenem	–	100.0%	100.0%	100.0%	66.7%	100.0%	100.0%	–	100.0%	100.0%
Meropenem	100.0%	100.0%	100.0%	100.0%	100.0%	100.0%	100.0%	100.0%	100.0%	100.0%
Sulfamethoxa-zole	0.0%	60.0%	40.0%	33.3%	100.0%	33.3%	66.7%	100.0%	50.0%	100.0%
Cefoperazone/sulbactam	100.0%	75.0%	100.0%	100.0%	66.7%	100.0%	100.0%	100.0%	100.0%	100.0%
Minocycline	0.0%	50.0%	0.0%	0.0%	33.3%	–	50.0%	100.0%	100.0%	100.0%
Doxycycline	0.0%	–	–	–	–	–	–	100.0%	–	–
Tigecycline	0.0%	100.0%	0.0%	0.0%	100.0%	0.0%	100.0%	100.0%	100.0%	100.0%
Nitrofurantoin	–	100.0%	0.0%	0.0%	–	0.0%	–	–	–	–
Amoxicillin/clavulanate	–	0.0%	0.0%	0.0%	–	–	0.0%		100.0%	100.0%
Ticacillin/clavulanate	50.0%	–	–	–	–	–	–	–	–	–

The antimicrobial agent in the antimicrobial susceptibility testing changes from year to year, so the sensitivity rate is calculated based on the number of bacteria that have actually tested the antimicrobial agent

## Discussion

The treatment of DNF is a major global challenge for healthcare workers [28]. Without prompt treatment with appropriate antibiotics and/or debridement of infected tissues, patients may develop toxic shock syndrome, leading to multiple organ failure and mortality. However, the result of bacterial susceptibility is not only lagging but also has a high false-negative rate, therefore, it has limited guiding significance for clinical antibiotic application [29]. Additionally, before the bacterial culture results are available, the choice of antibiotics is mainly based on the doctor’s experience and the severity of the infection [26]. Studies from different regions have demonstrated that there are different microbial compositions in diabetes mellitus with NF [17, 19, 21, 22, 30]. Moreover, there are globally very few studies on the bacterial distribution and drug susceptibility analyses of DNF, particularly regarding bacterial susceptibility. Our study reports the distribution of DNF pathogenic bacteria and analyzes the antimicrobial susceptibility to provide guidance for the treatment of DNF patients.

Type 1 NF, known as the polymicrobial form, is caused by more than two types of pathogenic bacteria and tends to occur in immunodeficient hosts or patients with chronic diseases, including diabetes [20, 31]. In our study, the proportion of patients with polymicrobial infection was 27.7%, which is quite different from the studies of Chen et al. [17] (81.4%) and Tan et al. [19] (39.7%), but similar to that of Cheng et al. [20] (26.2%). The reason for the difference may be that anaerobic bacteria were not tested in present study, and 72.3% of patients used antibacterial drugs before admission. Our results indicated that the DNF-causing bacteria were predominantly Gram-positive (61.3%), which is consistent with the published results [17, 19]. Although the main DNF-causing bacteria were Gram-positive bacteria, the microbial culture of type I NF can usually identify both aerobic and anaerobic microorganisms [20], thus the initial antibiotic treatment recommendations for DNF should be broad-spectrum coverage or combination therapy, including for Gram-positive and Gram-negative bacteria and anaerobes. In our results, *S. aureus* was the most frequently isolated bacteria, which is consistent with the study of Tan et al. [19], while in the study of Shaikh, it was *E. coli* [22], and in the study of Kumar et al., it was *β-hemolytic Streptococcus* and *E. coli* [30]. These pathogen differences may be due to regional differences, but also due to different bodily regions of the NF.

To our knowledge, there is no research reporting the MDR of DNF. DNF infection control is closely associated with the resistance of pathogenic bacteria, particularly in MDR infections. In our study, the proportion of MDR bacteria to the total cultured bacteria was similar to that in severe infection of DFUs reported by Hartemann-Heurtier et al. [32] (25.0%) and Li et al. [26] (33.8%). High-risk factors for MDR include poor blood sugar control, an ulcer size > 4 cm, frequent hospitalizations due to the same DFU, long duration of diabetic foot infection, long hospital stay, osteomyelitis, and long duration of antibiotic treatment [33]. The initial empirical antimicrobial therapy can be strengthened in patients with the above risk factors. In our results, 25% of the isolated *S. aureus* were MRSA, and it has been reported that the incidence of MRSA in monomicrobial NF has increased [34, 35], which requires clinical attention. *K. pneumoniae*, *P. mirabilis*, *M. morganii, E. coli*, and *E. cloacae* had a very high MDR ratio, which may be linked to the frequency of genes involved in the development of virulence and resistance [36], but the investigation of antimicrobial susceptibility for these bacteria should be improved.

Empirical antimicrobials are essential for DNF infection control. However, pathogenic bacteria in DNF patients may display resistance to the initial empirical antimicrobials. Of the total Gram-positive bacteria, MDR accounted for a relatively low proportion, and MRSA only accounted for 6.6% of the Gram-positive bacteria. Therefore, in the absence of a high risk for MRSA, DNF caused by Gram-positive bacteria can be treated with the antibacterial drugs levofloxacin, moxifloxacin, vancomycin, teicoplanin, tigecycline, and linezolid. However, if there is a high risk for MRSA, only vancomycin, teicoplanin, tigecycline, and linezolid are recommended. Our results indicate that Gram-positive cocci in DNF exhibit a high resistance to ciprofloxacin, clindamycin, penicillin G, tetracycline, and erythromycin, thus it is not recommended to use these drugs alone in the initial empiric antimicrobial therapy. Although the number of MDR Gram-negative bacteria accounted for more than half of the total number of Gram-negative bacteria, carbapenem-resistant bacteria and ESBL-producing Enterobacteria were less than 10%. Therefore, in addition to high-risk patients with carbapenem-resistant and ESBL-producing bacteria, DNF caused by Gram-negative bacteria can be treated with amikacin, piperacillin/tazobactam, cefoperazone/sulbactam, ceftazidime, cefepime, and carbapenems. Due to the high resistance rate to levofloxacin, ciprofloxacin, and ceftriaxone, we do not recommend these for the initial treatment. *P. aeruginosa* is naturally resistant to ertapenem, but it was one of the most common Gram-negative bacteria in our results, thus imipenem and meropenem are better than ertapenem for Gram-negative bacteria in DNF patients, which was consistent with Behzadi’s findings [37]. Although the bacteria isolated from DNF patients were mainly Gram-positive, while Gram-negative bacteria overall accounted for one-third of the total, DNF progressed rapidly and was critical, with the amputation rate as high as 31.7%, for which MDR accounted for a large proportion (28.2%). Therefore, for the initial empirical treatment, broad-spectrum antibacterials are still recommended. Because DNF is frequently associated with anaerobe infection [17], metronidazole or clindamycin is recommended if treatment for anaerobes is not included in the initial empiric antimicrobial spectrum.

### Limitations

There were limitations in this study. Firstly, 72.3% of DNF patients had used antibiotics when admitted to the hospital, which can lead to a decrease in the number of cultured bacteria; secondly, the majority of DNF patients are in serious condition when admitted, leading to potential selection bias; thirdly, the bacterial susceptibility test lacked anaerobic bacteria results.

## Conclusion

Our results indicate that the bacteria isolated from DNF patients are mainly Gram-positive bacteria. The initially experienced medication should fully consider the bacterial composition and proportion of MDR among the pathogens, and the high risk for amputation, and it is recommended to use broad-spectrum antibacterials. This study may help to determine the initial empirical clinical antibacterial and subsequent targeted antibacterial therapy for DNF patients.

## Data Availability

All data generated or analyzed during this study are included in this published article.

## Abbreviations

NF: Necrotizing fasciitis
DNF: Diabetic foot ulcers complicated by necrotizing fasciitis
DFU: Diabetic foot ulcer
IWGDF: International working group on the diabetic foot
MDR: Multi-drug resistant
ESBL: Extended-spectrum beta-lactamases
MRSA: Methicillin-resistant *Staphylococcus aureus*
